# 75. Utility of Fungal Blood Cultures in Portland, Oregon

**DOI:** 10.1093/ofid/ofab466.277

**Published:** 2021-12-04

**Authors:** Sujeet Govindan, Luke Strnad

**Affiliations:** Oregon Health & Science University, Portland, Oregon

## Abstract

**Background:**

Fungal blood cultures (fungal isolators) should be used, if at all, primarily for identification of mold infections. At our institution we noted patients having fungal blood cultures drawn in many other situations, including when the primary team was concerned for candida bloodstream infection. We sought to describe the utility of this practice and of fungal blood cultures in general.

**Methods:**

We retrospectively reviewed the results of fungal blood cultures for 2 years, from 3/1/2019-3/1/2021. We evaluated the number of episodes, culture results, whether there was a had prior bloodstream infection, and risk factors for fungal infection including renal replacement (RRT), ECMO, and immunosuppression (IS). Immunosuppression was defined as chronic systemic steroid use, recent receipt of high dose steroids within 2 weeks, history of organ transplantation, history of stem cell transplantation, hematologic malignancies, or receipt of a biologic agent.

**Results:**

187 fungal blood cultures were drawn in 143 patients - 80 cultures in 70 patients from 3/2019-3/2020 and 107 cultures in 73 patients from 3/2020-3/2021. Only 3 patients had positive fungal blood cultures:1 (*Candida krusei*) from 3/2019-3/2020 and 2 (*Candida albicans and Cyrptococcus neoformans*) from 3/2020-3/2021; in all 3 cases the organism also grew from standard blood culture isolators. From 3/2019-3/2020, 1/80 cultures were drawn from an individual on ECMO while 15/80 were drawn from individuals on RRT, and 32/80 were in a IS individuals. From 3/2020-3/2021, 45/107 cultures were drawn from an individual on ECMO, 24/107 were drawn in an individual on RRT, and 73/107 were drawn in a IS individuals. The majority of individuals in whom a fungal blood culture was drawn during 3/2020-3/2021 were individuals with COVID-19. Upon chart review most of the cultures were drawn due to concern for candidemia.

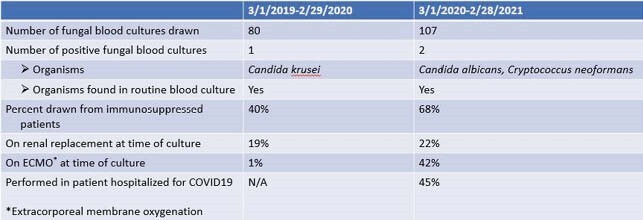

Results of fungal blood cultures drawn from 3/2019-3/2021 at OHSU

**Conclusion:**

Fungal blood cultures have an extremely low yield at our institution, with a 1.6% positivity rate over a 2 year period, and all of those cultures were detected by standard blood culture isolators. Most of these cultures were drawn in situations where this test has no utility. Furthermore, the test has limited utility to detect dimorphic and mold bloodstream infections. Restriction of this test may limit inappropriate use.

**Disclosures:**

**All Authors**: No reported disclosures

